# A pilot study of dietary fibres on pathogen growth in an *ex vivo* colonic model reveals their potential ability to limit vancomycin-resistant *Enterococcus* expansion

**DOI:** 10.20517/mrr.2022.14

**Published:** 2023-06-15

**Authors:** Ronan Strain, Tam T.T. Tran, Susan Mills, Catherine Stanton, R. Paul Ross

**Affiliations:** ^1^Food Biosciences Department, Teagasc Food Research Centre, Moorepark, Fermoy, Co. Cork P61C996, Ireland.; ^2^APC Microbiome Ireland, University College Cork, Co. Cork T12YT20, Ireland.; ^3^Microbiology Department, University College Cork, Co. Cork T12TP07, Ireland.

**Keywords:** Dietary fibre, gut microbiota, pathogens, colonisation resistance

## Abstract

**Aim**: Dietary fibre is important for shaping gut microbiota. The aim of this pilot study was to investigate the impact of dietary fibres on pathogen performance in the presence of gut microbiota.

**Methods**: In an *ex vivo* gut model, pooled faecal samples were spiked with a cocktail of representative gastrointestinal pathogens and fermented with yeast β-glucan for 24 hours, after which 16S rRNA amplicon sequencing and short-chain and branched-chain fatty acid (SCFA and BCFA) analyses were performed. In addition, oat β-glucan, arabinoxylan, yeast β-glucan, and galactooligosaccharides were each tested against individual representative pathogens and pathogen growth was assessed via qPCR. Glucose served as a control carbon source.

**Results**: Based on 16S rRNA amplicon sequencing, yeast β-glucan selected for higher proportions of *Bacteroides* (*P* = 0.0005, ~6 fold) and *Clostridia* (*P* = 0.005, ~3.6 fold) while species of *Escherichia/Shigella* (*P* = 0.021, ~2.8 fold) and *Lactobacillus* (*P* = 0.007, ~ 15.7-fold) were higher in glucose. Pathogen relative abundance did not differ between glucose and yeast β-glucan. In the absence of pathogens, higher production of BCFAs (*P* = 0.002) and SCFAs (*P* = 0.002) fatty acids was observed for fibre group(s). For individual pathogens, yeast β-glucan increased growth of *Escherichia coli*, *Salmonella typhimurium*, and *Listeria monocytogenes* (*P* < 0.05), arabinoxylan increased *S. typhimurium* (*P* < 0.05). Tested fibres decreased vancomycin-resistant *Enterococcus faecium* (*P* < 0.05), with yeast β-glucan causing a 1-log reduction (*P* < 0.01), while galactooligosaccharides decreased *L*. *monocytogenes* (*P* < 0.05).

**Conclusion**: Tested fibres differentially influenced the growth of pathogens, but yeast β-glucan could represent a dietary strategy to help limit vancomycin-resistant enterococci (VRE) expansion in the gut.

## INTRODUCTION

The gut microbiome is a diverse and metabolically active community consisting of more than 1,000 bacterial species, with total cell estimates of approximately ~3.9 × 10^13^ microbial cells^[1]^. These bacterial symbionts perform a broad range of functions, such as the digestion of complex dietary polysaccharides, production of vitamins and metabolites, interaction with and maintenance of the immune system, and defence against pathogens^[2]^. Factors including age, genetics, and lifestyle influence microbiome composition^[3]^. Furthermore, dietary components that are recalcitrant to digestion by host enzymes provide energy sources for bacterial growth and metabolism in the colon^[4]^. Thus, diet can also be considered a dominant selective force that drives microbiota community structure and function.

Dietary fibre is defined as non-digestible carbohydrates of ≥ 3 monomeric units naturally present in foods, but also includes isolated or synthetic counterparts that have been determined to provide physiological benefits^[5]^. Accordingly, many species of the gut microbiota are well equipped with an enzymatic repertoire that can convert these substrates into monosaccharides and their associated metabolites. The major end products of these reactions are short-chain fatty acids (SCFAs), which have favourable physiological consequences for the host, ranging from glucose or lipid homeostasis to tumour protection^[6]^. Intake of dietary fibre is widely considered to contribute to a more “homeostatic” diverse gut community^[7]^ through interactions among hundreds of bacterial species, driven primarily by cross-feeding and competition^[8]^.

The importance of the indigenous gut microbiota in protecting against enteric pathogens (termed “colonisation resistance”) has been realised as far back as the 1950s, when Bohnhoff *et al.* demonstrated that antibiotic treatment significantly increased murine susceptibility to *Salmonella* infection^[9]^. Similar mechanisms of colonisation resistance exist in humans, with antibiotic-mediated destruction of the microbiota leading to an expanse of opportunistic pathogens such as *Clostridioides difficile*^[10]^*.* Likewise, perturbations stemming from a diet absent of dietary fibre have been observed in mice^[11,12]^ with a concomitant increased risk of pathogen infection^[13,14]^. Interestingly, the reduced gut diversity and pathogen sensitivity can be mitigated through dietary interventions containing Microbiota-Accessible Carbohydrates (MACs)^[12,14]^. For example, certain dietary fibres have been shown to provide a protective effect against *Listeria monocytogenes* in animal models^[15]^. In this regard, the contribution of dietary fibre in influencing taxa that provide colonisation resistance against clinical pathogens is an emerging field of investigation.

Gut fermentation models have been used to study the changes in the microbiota in response to many interventions, including probiotics, prebiotics and pathogens, and the lack of a host means that there are fewer ethical concerns. In this pilot study, we employed the use of an *ex vivo* model of the human colon to study the effect of different dietary fibres [arabinoxylan, oat β-glucan, yeast β-glucan, and galacto-oligosaccharides (GOS)] on the gut microbiota and pathogen survival. To overcome individual bias, we used pooled faecal samples to represent the gut microbiota which was spiked with clinically relevant gastrointestinal pathogens as a specific cocktail or individually. For the specific pathogen cocktail (consisting of representative strains of *Citrobacter rodentium, Cronobacter sakazakii, Escherichia coli,* and *Salmonella typhimurium*), the effect of fibre on the gut microbiota and pathogen performance following 24-hour fermentation was assessed using 16s rRNA amplicon sequencing to help delineate the impact of microbiota changes on pathogen performance. For the individually spiked pathogens [consisting of representative strains of *E. coli*, *S. typhimurium*, *L. monocytogenes*, and *Enterococcus faecium* representing vancomycin-resistant *Enterococcus* (VRE)], quantitative PCR (qPCR) was used to directly investigate the impact of fibre on pathogen growth in the presence of the gut microbiota - with glucose as carbohydrate control in both cases. The production of SCFAs and branched-chain fatty acids (BCFAs) was also analysed for yeast β-glucan and the pathogen cocktail. This work originated from the Ph.D. dissertation of Ronan Strain^[16]^.

## METHODS

### Simulated human digestion of fibres

To replicate the digestion process in the upper gastrointestinal tract, 30 grams of crude-extract dietary fibre was subjected to a simulated gastric digestion process in order to produce a fibre substrate that would be similar to the one that enters the colon *in vivo*, which was adapted from a previous study^[17]^. This process represents three enzymatic phases of the digestion process; oral, gastric and intestinal phase. In the oral phase, each experimental dietary fibre was mixed in a 50:50 (w/v) ratio with simulated salivary fluid [KCl 15.1 mmol·L^-1^; KH_2_PO_4_ 3.7 mmol·L^-1^; NaHCO_3_ 13.6 mmol·L^-1^; MgCl_2_(H_2_O)_6_ 0.15 mmol·L^-1^; (NH_4_)_2_CO_3_ 0.06 mmol·L^-1^; adjusted to pH 7.0] and human salivary α-amylase (Sigma, Ireland) (pre-warmed to 37 ^o^C) was added to achieve 75 U·mL^-1^, followed by addition of CaCl_2_ to achieve 0.75 mM, and the enzyme digestion was allowed to proceed for 2 minutes at 37 ^o^C. The resulting digested mixture was subjected to simulated gastric digestion with the mixture added in a 50:50 (v/v) ratio to simulated gastric fluid [KCl 6.9 mmol·L^-1^; KH_2_PO_4_ 0.9 mmol·L^-1^; NaHCO_3_ 25 mmol·L^-1^; NaCl 47.2 mmol·L^-1^; MgCl_2_(H_2_O)_6_ 0.1 mmol·L^-1^; (NH_4_)_2_CO_3_ 0.5 mmol·L^-1^; adjusted to pH 3.0]. Porcine pepsin (Sigma) was added to achieve 2,000 U·mL^-1^ and CaCl_2_ added to achieve 0.075 mM. pH was adjusted to 3.0 with 1 M HCl and the mixture was incubated for 2 hours at 37 ^o^C in a shaking incubator. The mixture product from the gastric phase was then subjected to simulated intestinal digestion using a 50 : 50 (v/v) simulated intestinal fluid [KCl 6.8 mmol·L^-1^; KH_2_PO_4_ 0.8 mmol·L^-1^; NaHCO_3_ 85 mmol·L^-1^; NaCl 38.4 mmol·L^-1^; MgCl_2_(H_2_O)_6_ 0.33 mmol·L^-1^; adjusted to pH 7.0]. A pancreatin (Sigma) solution was prepared in advance using the simulated intestinal fluid as a solute and this was added to the mixture at a final concentration of 100 U·mL^-1^. Bile salts were added at a final concentration of 10 mM, CaCl_2_ at a final concentration of 0.3 mM and the pH adjusted to 7.0 prior to 2-hour digestion at 37 ^o^C. The end-product was dialysed in a 1 KDa dialysis membrane to account for absorption in the small intestine, and subsequently lyophilised and stored at -20 ^o^C.

### Faecal sample collection and processing

Stool samples were collected from consenting healthy volunteers (*n* = 6) under the approval of the Clinical Research Ethics Committee (CREC) of the Cork Teaching Hospitals according to the study protocol APC 055. The healthy volunteers were not taking prescribed medications and had not taken antibiotics in the previous 3 months. A standardised faecal inoculum was prepared as described previously^[18]^ by pooling 60 g of each (*n* = 6) subject’s faecal sample. The standardised faecal slurry was immediately frozen in aliquots (100 mL) at -80 ^o^C, and on the day of the fermentation experiment, it was thawed at 37 ^o^C for 1 hour prior to use.

### Gastrointestinal pathogens

All the test pathogens were obtained from the DPC culture collection (Teagasc Moorepark, Ireland). *Cit. rodentium* (DPC 6470; ICC168), *C. sakazakii* (DPC 6090; ATCC 12868) and *E. coli* (DPC 6054; P1432) were routinely grown aerobically overnight at 37 ^o^C in Luria-Bertani broth (Merck Millipore, Ireland). *S. typhimurium* LT2 (DPC 6048; ATCC 700720), *E. faecium* TX16 (APC 852; ATCC BAA-472) and *L. monocytogenes EGDe* (APC 154; DPC 6554; ATCC BAA-679) were grown aerobically overnight at 37 ^o^C in Brain Heart Infusion broth (Merck Millipore). On the day of faecal fermentation, overnight cultures of pathogens were subcultured into fresh aliquots of their corresponding medium and grown to mid-log phase (OD600 - 0.1), and cells were harvested by centrifugation at 4,000 × g at 4 ^o^C for 15 min and resuspended in fresh faecal fermentation medium (see *Ex vivo* model of the distal colon) to give a final concentration of 1 × 10^5^ cfu/mL of each pathogen. The four-strain pathogen cocktail containing *Cit. rodentium, C. sakazakii, E. coli,* and *S. typhimurium* was prepared on the day of the fermentation experiment.

### *Ex vivo* model of the distal colon

A basal no-carbon faecal fermentation medium [contents: g·L^-1^; peptone water, 2.0; yeast extract, 2.0; NaCl, 0.1; K_2_HPO_4_, 0.04; KH_2_PO_4_, 0.04; CaCl_2_.6H_2_0, 0.01; MgSO_4_.7H_2_0, 0.01; NaHCO_3_, 2.0; Tween 80, 2 mL; hemin (dissolved in three drops 1 M NaOH), 0.05; Vitamin K_1_, 10 mL; cysteine-HC1, 0.5; bile salts, 0.5] was prepared as described previously^[19]^ overnight in an anaerobic chamber. The Applikon MicroMatrix (Applikon Biotechnology, The Netherlands) *ex vivo* model was employed to model the human distal colon as described previously^[20]^. Each vessel contained 4.75 mL of faecal fermentation medium combined with 0.25 mL faecal slurry (5% v/v) and 1% w/v test carbohydrate. Thus, to begin the experiment, 2 mL of a T_0_ baseline sample containing 4.75 mL of the appropriate fermentation medium and 0.25 mL faecal slurry was centrifuged at 16,000 × g for 15 mins at 4 ^o^C and the pellet and supernatant immediately frozen at -80 ^o^C. For the impact of yeast β-glucan on the pathogen cocktail, the 24-well MicroMatrix cassette was divided into four groups, *n* = 6 for each group, consisting of (1) glucose with no pathogen added (GN), (2) yeast β-glucan with no pathogen added (YN), (3) glucose with pathogen cocktail added (GP), (4) yeast β-glucan with pathogen cocktail added (YP).

For the impact of oat β-glucan, arabinoxylan, yeast β-glucan on the individual pathogens, *E. coli*, *S. typhimurium*, and VRE, each 24-vessel plate consisted of the three test fibres and glucose as control: Glucose (*n* = 3); Arabinoxylan (*n* = 3); Oat fibre (*n* = 3) and Yeast β-Glucan (*n* = 3); spiked with a test pathogen. This approach enabled two pathogen strains to be studied in triplicate for each fibre on a run. For *L. monocytogenes*, a separate 24-well plate was used and GOS (*n* = 3) and cellulose (*n* = 3) were also included. The MicroMatrix fermentation experiment was allowed to run for 24 hours with the temperature kept constant at 37 ^o^C, pH at 6.8, anaerobiosis maintained by the addition of N_2_ gas and orbiter set at 300 rpm. Following 24 hours of fermentation, 2 mL of each vessel fermentate were collected and centrifuged at 16,000 × g for 15 min at 4 ^o^C and the pellet and supernatant were immediately frozen at -80 ^o^C.

### DNA extraction

Bacterial DNA was extracted using the Repeated Bead Beating (RBB) plus column method adapted from Yu and Morrison^[21]^, and extracted DNA was washed with buffers AW1 and AW2 from Qiagen Blood & Tissue Kit (Qiagen, Hilden, Germany), following manufacturer’s instructions. DNA was extracted from the pellets collected from baseline (T_0_) and T_24_ fermentation vessels.

To generate standard curves for qPCR, pure genomic DNA of pathogens was extracted using GenElute™ Bacterial Genomic DNA Kit (Sigma) according to the manufacturer’s instructions.

### Bacterial 16S rRNA amplicon sequencing

The V3-V4 region of the 16S rRNA gene was amplified according to the Illumina 16S rRNA amplicon metagenomics sequencing protocol, creating a 460bp amplicon using the forward primer 5’TCGTCGGCAGCGTCAGATGTGTATAAGAGACAGCCTACGGGNGGCWGCAG and reverse primer 5’GTCTCGTGGGCTCGGAGATGTGTATAAGAGACAGGACTACHVGGGTATCTAATCC^[22]^. Indexed products were cleaned with AMPure XP beads (Beckman Coulter, Indiana, US), quantified with Qubit dsDNA HS Assay kit (Life technologies, California, US) and subsequently pooled in an equimolar fashion. Samples were sequenced on the Illumina MiSeq Sequencing platform (Clinical Microbiomics, Denmark) using a 2 × 250 cycle kit (Illumina).

### Analysis of sequencing data

Illumina MiSeq reads were analysed using the Quantitative Insights into Microbial Ecology (QIIME) v.1.9.1 and uSearch v.8.1 software as described previously^[23]^. Paired-end reads were merged using FLASH v.1.2.8 and adaptors were removed using cutadapt v.1.8.3. Sequences for each amplicon were clustered at 97% similarity into operational taxonomic units (OTUs). An OTU table was obtained using uSearch. Classification of representative sequences for each OTU was carried out using mothur v.1.36.1. against the 16S rRNA gene reference of Ribosomal Database Project database trainset 14. Scripts for generation of alpha and beta diversity can be found in the study by Tran *et al.*^[23]^.

### Quantitative PCR

Specific primers [Table 1] were used targeting different genes in each bacterium [Table 1] that ensured specificity. For *E. faecium* TX16, primers targeting the 16S rRNA gene were designed using PrimerBlast^[24]^, while the remaining primer pairs were publicly available [Table 1]. To determine the optimal annealing temperature for qPCR primers [Table 1], primers were tested against genomic DNA isolated from the appropriate pathogen. The master mix was made to cover 24 reactions, each containing 12.5 µL Biomix red (Bioline), 10 µL mqH_2_O, 1 µL of 10 µM forward primer, 1 µL of 10 µM reverse primer and 0.5 µL gDNA from fermentate per reaction. PCR was performed on an Applied Biosystems 2720 thermal cycler (Life Technologies). The annealing temperature gradient was based on the average Tm of the forward and reverse primers minus 5 ^o^C; this value provided the median temperature over which gradient PCR was performed. No-template controls consisting of 0.5 µL mqH_2_0 in place of gDNA were run in order to eliminate temperatures under which primer dimers were potentially formed. Optimal annealing temperatures were chosen based on the vividness of the band of the test samples, and the absence of primer dimers in the no-template controls**.**

**Table 1:** Primers used for qPCR

**Primer Name**	**Sequence (5’-3’)**	**Product Size (bp)**	**AT (^o^C)**	**Target Gene (Organism)**	**Gene Accession #** **(Organism Accession #)**	**Reference**
EcH7F EcH7R	GCGCTGTCGAGTTCTATCGAGCCAACGGTGACTTTATCGCCATTCC	625	65	H7 antigen*(E. coli p1432)*	Not submitted	[50]
Stm4497F Stm4497R	GGAATCAATGCCCGCCAATGCGTGCTTGAATACCGCCTGTC	542	66	STM4497 *(S. typhimurium LT2)*	NP_463356.1 (NC_003197.2)	[51]
PrfAF PrfAR	GATACAGAAACATCGGTTGGCGTGTAATCTTGATGCCATCAGG	274	60	PrfA *(L. monocytogenes* EDG-e)	6EUT_B (NC_003210.1)	[52]
Ef16SF Ef16SR	GCTTCTTTTTCCACCGGAGCCTGCCTCCCGTAGGAGTTTG	305	55	16S *(E. faecium TX16)*	HMPREF0351_r10001 (NC_017960.1)	This study

The spiked pathogens (*E. coli, S. typhimurium, E. faecium* and *L. monocytogenes*) were then detected in the 24-hour fermentates using qPCR. Detection of pathogens was achieved using absolute quantification on a LightCycler® 96 and software (Roche, Basel, Switzerland) according to the manufacturer’s instructions. Fibre and pathogen combinations were performed in triplicate in the same run. Quantitation cycle (Cq) values of each sample were compared with a standard curve (in duplicate) following the MIQE guidelines^[25]^. The standard curve was generated by diluting known concentrations of pathogen genomic DNA containing a known copy number of target gene in a five-fold serial dilution (10^7^ - 10^2^ copies/µL). No-template controls were included in duplicate consisting of mqH_2_O in place of DNA. An additional negative control consisting of a faecal metagenomic DNA sample without pathogen was included in each run to ensure non-specific amplification of primers. A T_0_ fermentate sample was included in each run, consisting of fermentation media, faecal inocula and spiked pathogen. The qPCR data were expressed as gene copy number per µL of fermentate. The Efficiency of primers (E) was estimated from the slope according to the equation: E = 10 (-1/slope)^[26]^. The theoretical maximum PCR efficiency of 100% indicates the amount of product that doubles with each amplification cycle [Table 1]. qPCR conditions for each reaction (10 µL) were as follows: 5 µL KAPA SYBR® fast qPCR Mastermix (Kapa Biosystems), 1 µL of 10 µM forward primer, 1 µL of 10 µM reverse primer, 3.8 µL mqH_2_O, 1 µL DNA. Reactions were incubated in a Lightcycler® 96 for 5 mins at 94 ^o^C, then 40 cycles of 94 ^o^C for 30s, AT ^o^C [Table 1] for 30 s and 72 ^o^C for 30 s. The ability of a substrate to selectively influence the growth of a spiked pathogen was determined by comparing incubations with the glucose control.

### Short and branched chain fatty acid analysis

A 2 mL aliquot of fermentation liquid was syringe-filtered (0.2 µM) to remove bacterial debris and 1 mL of this was subsequently subjected to gas chromatography as described previously^[27]^. Briefly, 1 mL of filtered faecal fermentate was mixed with 2-Ethylbutyric acid (Sigma) as an internal standard. SCFAs were measured by gas chromatography using a Varian 3500 GC flame-ionization system fitted with a Zebron ZB-FFAP column (Phenomenex, UK). Conditions for chromatography were as follows: GC oven temperature was pre-heated to 50 ^o^C for 30 s, then incremented stepwise by 10 ^o^C/min until a temperature of 140 ^o^C was reached. This process was followed by 20 ^o^C/min increments until a temperature of 240 ^o^C was reached and then held for 5 min. The injector temperature was set at 240 ^o^C and the detector at 300 ^o^C. Helium gas was used as the carrier at a flow rate of 1.3 mL/min^-1^. A standard curve was constructed based on increasing concentrations of SCFA and BCFA solutions (Sigma, Ireland). The Varian Star Chromatography Workstation v6.0 software was used to integrate peaks from the test samples and the concentrations of SCFA and BCFA were calculated using the linear regression equations (*R*^2^ ≥ 0.999) from the inputted standard curve. SCFA and BCFA standards were included on the run to check for calibration. The data were presented as mMol.

### Statistical analysis

For 16s rRNA amplicon sequencing data, statistical analyses were performed using the software package R v.3.5.1. Kruskal-Wallis *H* test with Dunn’s multiple comparison test was performed for significant differences in alpha diversity and relative abundances of each OTU. A Benjamini-Hochberg correction was employed to correct for *P* values to control false discovery rates. The permutational multivariate ANOVA (PERMANOVA) analysis was used to determine significant differences in beta diversity. For SCFAs, data normality was assessed using the Shapiro-Wilk test using SPSS Version 22.0. Differences in total SCFAs and BCFAs were analysed using the univariate ANOVA with Bonferroni correction. For qPCR data, one-way ANOVA analysis with Dunnett’s post-hoc test was carried out, comparing the fibre treatments with the glucose control. Statistical analysis was performed on SPSS (IBM) and visualised using GraphPad Prism (GraphPad software). *P* < 0.05 was considered to be statistically significant.

## RESULTS

### 16s rRNA amplicon sequencing - diversity analyses and dominant taxa in the presence of glucose and yeast β-glucan with and without pathogen cocktail

#### Diversity analyses

**Figure 1:**
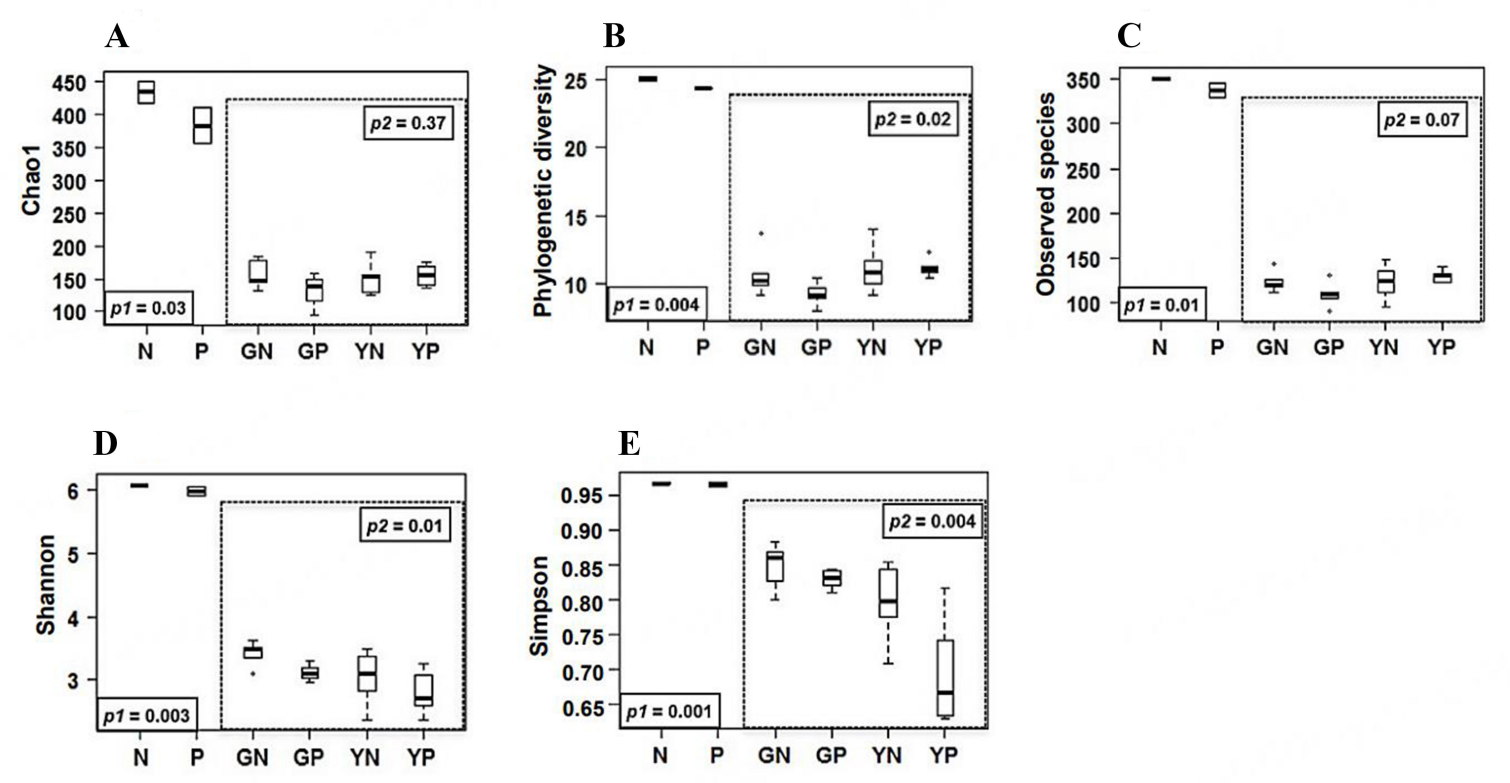
Alpha diversity measured by (A) Chao1, (B) Phylogenetic diversity, (C) Observed species, (D) Shannon and (E) Simpson indices is plotted for baseline inocula, no-pathogen (N), pathogen (P), 24-hour glucose no-pathogen (GN), 24-hour glucose pathogen (GP), 24-hour yeast β-glucan no-pathogen (YN), 24-hour yeast β-glucan pathogen (YP). The line inside the box represents the median, the boxes represent the interquartile range, and the whiskers represent the range. *P* values were calculated from the Kruskal-Wallis test; *P*1 when comparing inocula and 24-hour fermentation groups; *P*2 when comparing within fermentation groups.

High-throughput paired-end sequencing of the V3 and V4 region of the 16S rRNA gene was performed on the faecal fermentation samples using the Illumina MiSeq platform. Alpha diversity (expressed as the number of species or richness present in a group of samples) indices (Chao1; Phylogenetic diversity; Observed species; Shannon; Simpson) were determined and p values were calculated from the Kruskal-Wallis test [Figure 1A-E]. Fermentation with yeast β-glucan or glucose in the presence or absence of the pathogen cocktail consisting of representative strains of *Cit. rodentium, C. sakazakii, E. coli,* and *S. typhimurium* decreased alpha diversity indices between baseline (T_0_) and 24 hours after fermentation (T_24_). All the diversity indices were significant when comparing the baseline (T_0_) and the fermentation condition groups, indicated by the *P*1 value; Chao1 *P* = 0.03, Phylogenetic diversity *P* = 0.004, Observed species *P* = 0.01, Shannon *P* = 0.003, Simpson *P* = 0.001. When comparing between the fermentation groups, the alpha diversity indices Shannon *P* = 0.01, Simpson *P* = 0.004 and Phylogenetic diversity *P* = 0.02 were the only indices found to be significant, indicated by the *P*2 value.

**Figure 2:**
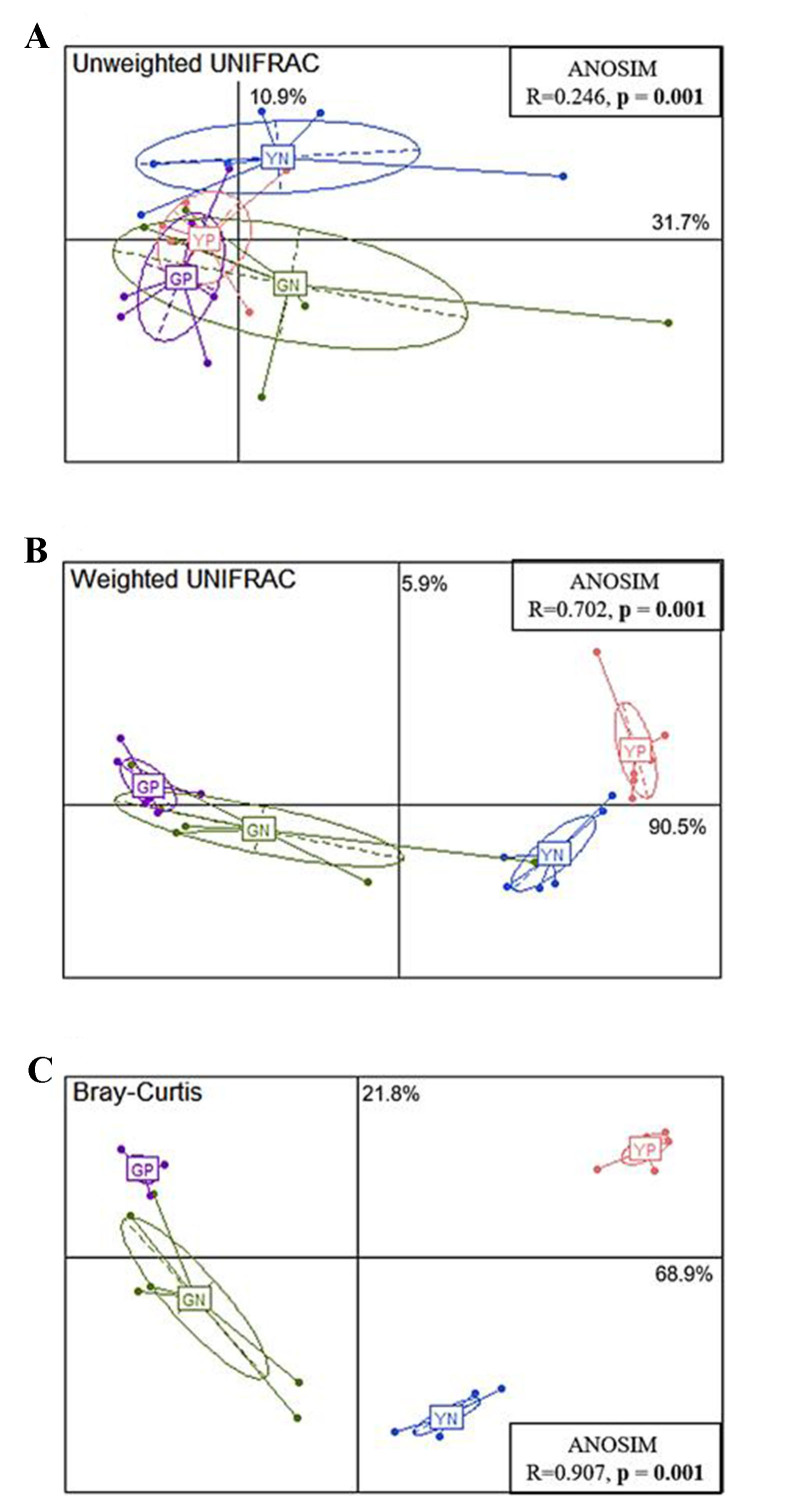
Principal Coordinate Analysis (PCoA) plots for (A) unweighted UNIFRAC, (B) weighted UNIFRAC and (C) Bray-Curtis distances for the four fermentation groups; 24-hour glucose no-pathogen (GN), 24-hour glucose pathogen (GP), 24-hour yeast β-glucan no-pathogen (YN), 24-hour yeast β-glucan pathogen (YP). The significant differences between groups were calculated by analysis of similarity (ANOSIM) tests.

**Figure 3:**
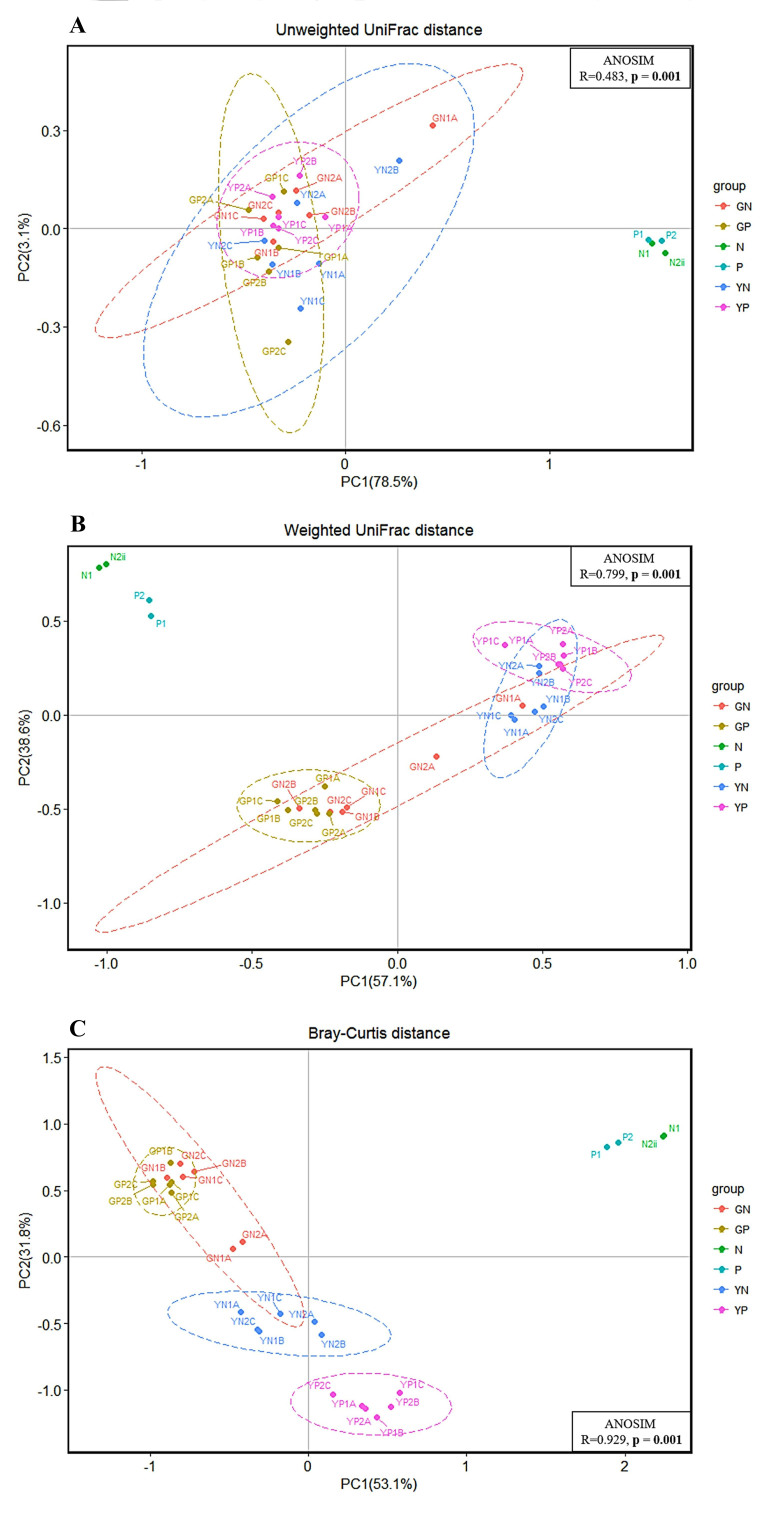
Principal Coordinate Analysis (PCoA) plots for (A) unweighted UNIFRAC (B) weighted UNIFRAC, and (C) Bray-Curtis metrics for the four fermentation groups with inocula included; inocula no-pathogen (N), inocula pathogen (P), 24-hour glucose no-pathogen (GN), 24-hour glucose pathogen (GP), 24-hour yeast β-glucan no-pathogen (YN), 24-hour yeast β-glucan pathogen (YP). The significant differences between groups were calculated by analysis of similarity (ANOSIM) tests.

Principal Coordinate Analysis (PCoA) of the fermentation groups using unweighted UNIFRAC, weighted UNIFRAC, and Bray-Curtis can be found in Figure 2A-C. These account for 31.7%, 90.5%, and 68.9% of the variation explained by the first principal coordinate, respectively. The significant differences between groups were calculated by analysis of similarity (ANOSIM) tests. UNIFRAC measures the phylogenetic differences, i.e., evolutionary differences between groups of samples, whereas Bray-Curtis dissimilarity measures the differences in species populations. Weighted UNIFRAC incorporates the relative abundances of species when calculating the differences in phylogenetic branch lengths, whereas unweighted UNIFRAC does not consider relative abundances; therefore, making this measure useful for low-abundance species. The PCoA plots [Figure 3A-C] with the baseline included (T_0_) illustrate a clear separation or decrease in microbial diversity between the T_0_ and T_24_ fermentation groups. The larger separation in the weighted UNIFRAC PCoAs compared with unweighted UNIFRAC would explain that there is a wider range of low-abundance species that benefit depending on the carbon source.

#### Taxonomic analyses

**Figure 4:**
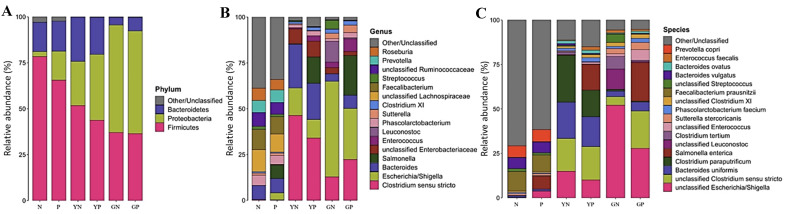
Mean relative abundances of taxonomic groups at phylum (A), genus (B), and species (C) levels for the four fermentation groups; 24-hour glucose no-pathogen (GN), 24-hour glucose pathogen (GP), 24-hour yeast β-glucan no-pathogen (YN), 24-hour yeast β-glucan pathogen (YP). Inocula groups (N, no pathogens and P, pathogen spiked). Graphs represent the means of 6 replicates.

At the phylum level, the fermented faecal slurries were dominated predominantly by Firmicutes, Proteobacteria, and Bacteroidetes [Figure 4A]. Comparing the yeast β-glucan (YN) and glucose (GN) faecal slurries in the absence of the pathogen cocktail revealed that Bacteroidetes (YN = 24.14%; GN = 4.26%; *P* = 0.0004) and Firmicutes (YN = 51.75%; GN = 37.07%; *P* = 0.019) were higher in the fibre-treated groups. Proteobacteria were capable of metabolising glucose more efficiently than yeast β-glucan (YN = 24%; GN = 58.52%; *P* = 0.007). Low abundant members of the Fusobacteria phylum were higher in the glucose-faecal slurry in the presence of the pathogen cocktail (GP) relative to the yeast β-glucan-faecal slurry in the presence of the cocktail (YP) (GP = 0.1%; YP = 0.01%; *P* = 0.034).

At the genus level [Figure 4B], the following genera were higher in the YN group compared with the glucose controls (GN), *Bacteroides* (YN = 23.85%; GN = 4.08%; *P* = 0.0005), *Clostridium sensu stricto* (YN = 46.34%; GN = 12.81%; *P* = 0.005) and unclassified *Enterobacteriaceae* (YN = 8.14%; GN = 3.36%; *P* = 0.043). Unclassified *Enterobacteriaceae* was the only main genus significantly higher in the fibre group than control when the pathogens were added (YP = 8.64%; GP = 2.16%; *P* = 0.01). The genera that performed better in the presence of glucose compared with fibre were *Escherichia/Shigella* (YN = 14.96%; GN = 52.13%; *P* = 0.011), *Enterococcus* (YN = 0.61%; GN = 3.05%; *P* = 0.035), *Leuconostoc* (YN = 0.04%; GN = 11.46%; *P* = 0.017) and *Sutterella* (YN = 0.49%; GN = 2.92%; *P* = 0.007). When the pathogens were added, higher *Escherichia/Shigella* (YP = 10.07%; GP = 27.86%; *P* = 0.021) and *Sutterella* (YP = 0.25%; GP = 4.04%; *P* = 0.001) were observed in the glucose treated groups.

At species level (Figure 4C - approximate predictions since 16S rRNA amplicon profiling approach does not allow precise taxonomical identification), six species of *Bacteroides* were significantly higher in the fibre treated groups; three of these were low-abundance members displaying minimally higher relative abundances [*Bacteroides intestinalis* (YN = 0.04%; GN = 0.01%; *P* = 0.032); *Bacteroides salyersiae* (YN = 0.23%; GN = 0.02%; *P* = 0.015); *Unclassified Bacteroides* (YN = 0.42%; GN = 0.04%; *P* = 0.008)]. One species performed much better in the presence of fibre compared with glucose: *Bacteroides uniformis* (YN = 20.41%; GN = 3.15%; *P* = 0.006) and two other species to a lesser extent, *Bacteroides ovatus* (YN = 1.6%; GN = 0.5%; *P* = 0.028) and *Bacteroides vulgatus* (YN = 0.95%; GN = 0.2%; *P* = 0.007). A species of *Clostridium paraputrificum* performed comparatively very well in the fibre groups (YN = 26.38%; GN = 0.94%; *P* = 0.015) and in the presence of pathogens (YP = 14.99%; GP = 0.54%; *P* = 0.012). An unclassified *Clostridium sensu stricto* (YN = 18.42%; GN = 4.77%; *P* = 0.043) was significantly higher in the fibre groups but not in both carbohydrate sources in the presence of pathogens (YP = 18.76%; GP = 21.0%; *P* = 0.461). An unclassified *Enterobacteriaceae* was significantly higher in the fibre groups (YN = 8.14%; GN = 3.36%; *P* = 0.043), including when spiked with pathogens (YP = 8.64%; GP = 2.16%; *P* = 0.01).

Two unclassified species were higher in the glucose groups compared with the fibre groups; an unclassified *Enterococcus* (YN = 0.2%; GN = 1.47%; *P* = 0.04) and an unclassified *Leuconostoc* (YN = 0.04%; GN = 11.46%; *P* = 0.017) (YP = 0.002%; GP = 0.54%; *P* = 0.007). *Sutterella stercoricanis* was significantly higher in the glucose groups with pathogens (YP = 0.19%; GP = 4.03; *P* = 0.001) and without pathogens (YN = 0.47%; GN = 2.91%; *P* = 0.013) compared with the respective fibre groups. An unclassified *Escherichia/Shigella* was significantly higher in the glucose groups (YN = 14.96%; GN = 52.13%; *P* = 0.011) (YP = 10.07%; GP = 27.26%; *P* = 0.021) in the presence or absence of pathogens compared with the respective fibre groups. There were no significant differences in pathogen relative abundances between fibre and control.

### Short chain and branched chain fatty acid analysis in the presence of glucose and yeast β-glucan and with and without pathogen cocktail

**Figure 5:**
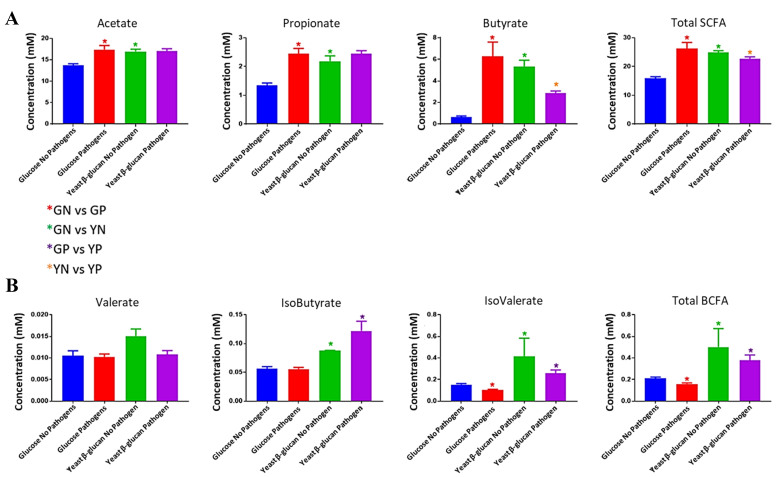
Short chain (A) and branched chain (B) fatty acid analysis for the four fermentation groups following 24-hour fermentation. 24-hour glucose no-pathogen (Blue), 24-hour glucose pathogen (Red), 24-hour yeast β-glucan no-pathogen (Green), 24-hour yeast β-glucan pathogen (Purple). *P < 0.05. SCFA: Short-chain fatty acid; BCFA: branched-chain fatty acid.

SCFAs are produced by catabolism of dietary carbohydrates by gut bacteria. Concentrations of SCFAs were compared for the four fermentation conditions to determine differences in microbiota production of SCFAs by carbohydrate source and presence of pathogens [Figure 5A]. When comparing the GN and GP groups, concentrations of acetate (GN = 13.65 mM, GP = 17.43 mM, *P* = 0.002), propionate (GN = 1.34 mM, GP = 2.45 mM, *P* = 0.002) and butyrate (GN = 0.65 mM, GP = 6.29 mM, *P* = 0.002) were significantly higher in the pathogen added group, suggesting that the spiked pathogens contributed to the production of SCFAs, through fermentation of glucose. With respect to the production of the BCFAs [Figure 5B], isovalerate (GN = 0.15 mM, GP = 0.1 mM, *P* = 0.002) and total BCFAs (GN = 0.22 mM, GP = 0.17 mM, *P* = 0.009) were significantly lower when the pathogens were added. Comparisons of the YN and YP groups showed that butyrate (YN = 5.32 mM, YP = 2.86 mM, *P* = 0.004) and total SCFAs (YN = 24.46 mM, YP = 22.29 mM, *P* = 0.041) were significantly lower in the pathogen added group, suggesting spiking of pathogens is having a negative effect on the fibre-degrading and butyrate-producing commensal bacteria. With regard to BCFA concentrations, there was no significant effect on the production of BCFAs in the yeast β-glucan groups when pathogens were added.

In terms of carbohydrate source, comparisons of the GN and YN groups showed significantly higher concentrations of SCFAs in the YN group [Figure 5A]: acetate (GN = 13.65 mM, YN = 16.95 mM, *P* = 0.002), propionate (GN = 1.34 mM, YN = 2.19 mM, *P* = 0.002), butyrate (GN = 0.65 mM, YN = 5.32 mM, *P* = 0.002) and total SCFAs (GN = 15.64 mM, YN = 24.26 mM, *P* = 0.002). Similarly, significantly higher concentrations of BCFAs [Figure 5B] were observed in the fibre group when comparing GN and YN; isobutyrate (GN = 0.056 mM, YN = 0.087 mM, *P* = 0.002), isovalerate (GN = 0.15 mM, YN = 0.41 mM, *P* = 0.002) and total BCFA (GN = 0.22 mM, YN = 0.3 mM, *P* = 0.002). Interestingly, the pathogen-spiked fibre groups had higher concentrations of isobutyrate (GP = 0.055 mM, YP = 0.122 mM, *P =* 0.041), isovalerate (GP = 0.1 mM, YP = 0.25 mM, *P* = 0.002) and total BCFA (GP = 0.17 mM, YP = 0.39 mM, *P* = 0.004), suggesting that the pathogens are contributing to the production of BCFA from fibre.

### Quantification of individual representative pathogens in the presence and absence of dietary fibres by qPCR

#### E. coli

**Figure 6:**
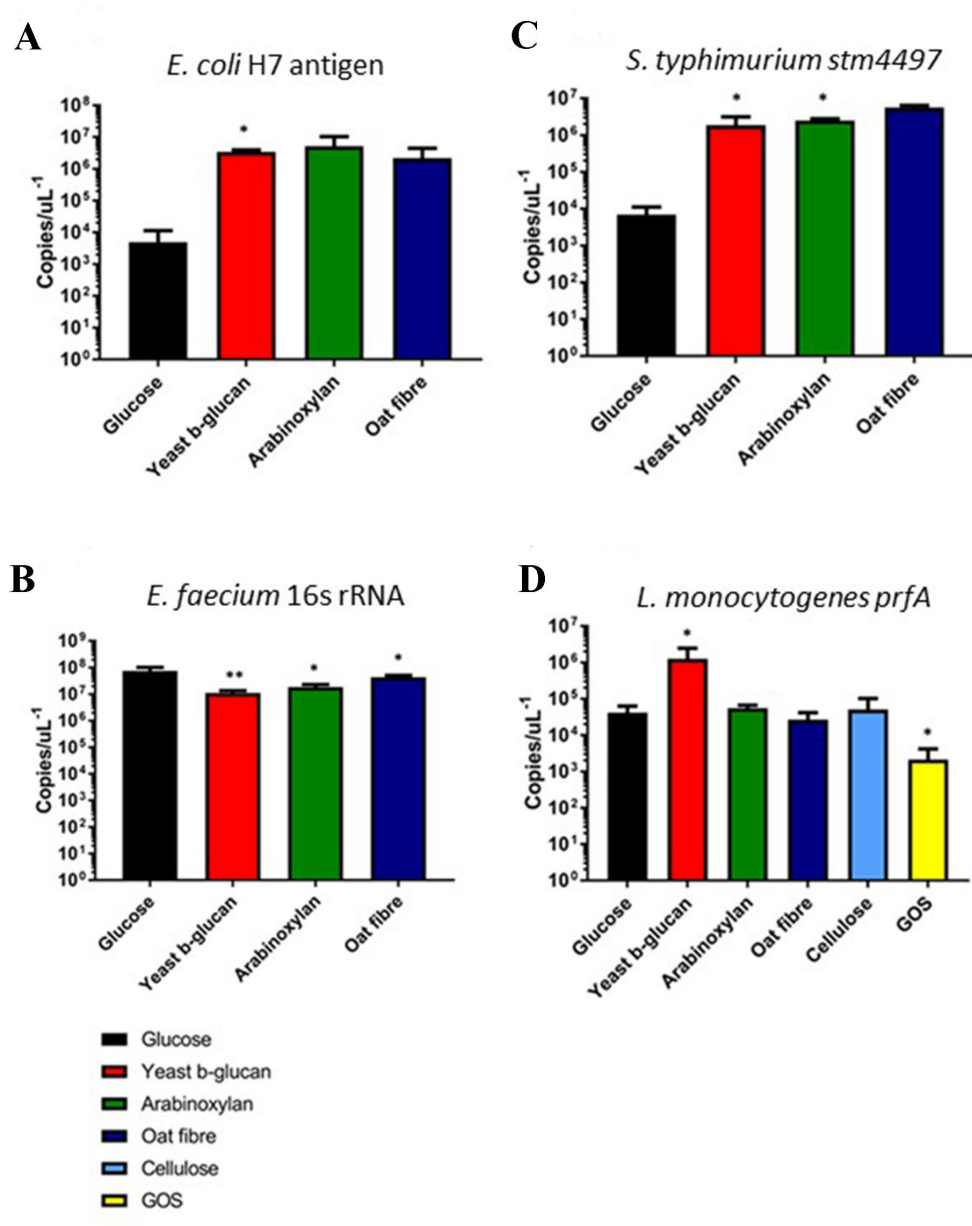
Gene copy numbers (log_10_ copy numbers per µL of fermentation effluent) for *E. coli* (A), *S. typhimurium* (B), vancomycin-resistant *E. faecium* (C), and *L. monocytogenes* (D) after 24-hour faecal fermentation with the pooled faecal inocula and test fibres as determined by qPCR. *Significant difference (*P* < 0.05) when comparing test carbohydrate with glucose control.

Growth of *E. coli* in the presence of glucose was limited, with a mean of 2.63 × 10^3^ copies/µL [Figure 6A]. Quantities in the fibre groups were 1.85 × 10^6^ copies/µL for yeast β-glucan, 3.57 × 10^6^ copies/µL for arabinoxylan, and 5.89 × 10^6^ copies/µL for oat fibre. One-way ANOVA with Dunnett’s test revealed a statistical significance when comparing the yeast β-glucan (*P* < 0.05) group with the glucose control. The other fibres tested were not significant.

#### S. typhimurium

Similar to *E. coli*, the growth of *S. typhimurium* was limited in the glucose control at 2.33 × 10^3^ copies/µL [Figure 6B]. Quantities in the fibre groups were 6.15 × 10^7^copies/µL for yeast β-Glucan, 8.35 × 10^7^ copies/µL for arabinoxylan, and 1.86 × 10^8^ copies/µL for oat fibre. One-way ANOVA with Dunnett’s test revealed statistical significance in the yeast β-glucan and arabinoxylan (*P* < 0.05) groups compared to glucose.

#### E. faecium TX16

The glucose control resulted in the highest *E. faecium* growth at 1.48 × 10^8^ copies/µL [Figure 6C]. Quantities in the fibre groups were 2.24 × 10^7^ copies/µL for yeast β-Glucan, 3.66 × 10^7^ copies/µL for arabinoxylan, and 8.71 × 10^7^ copies/µL for oat fibre. One-way ANOVA with Dunnett’s test revealed statistically significant differences in the yeast β-glucan (*P* < 0.01), arabinoxylan (*P* < 0.05) and oat fibre (*P* < 0.05) groups compared to glucose.

#### L. monocytogenes

Additional carbohydrates were tested in this run, namely GOS and cellulose [Figure 6D]. The glucose control resulted in concentrations of 3.14 × 10^4^ copies/µL. Quantities in the fibre groups were 1.25 × 10^6^ copies/µL for yeast β-Glucan, 7.20 × 10^4^ copies/µL for arabinoxylan, 2.15 × 10^4^ copies/µL for oat fibre, 7.58 × 10^4^ copies/µL for cellulose, and 4.59 × 10^3^ copies/µL for GOS. One-way ANOVA with Dunnett’s test revealed a significant difference in both the yeast β-glucan and GOS groups compared to the glucose control (*P* < 0.05).

## DISCUSSION

Dietary fibre has long been accepted as providing health benefits, such as normalising bowel movements and lowering blood cholesterol^[28,29]^. In this study, we investigated the impact of dietary fibres on pathogen performance in an *ex vivo* gut model using two different molecular approaches, 16S rRNA amplicon sequencing and qPCR.

16S rRNA amplicon sequencing provides culture-independent species-level taxonomic resolution^[30]^ and thus enabled us to investigate fibre-mediated changes in the gut microbiota and their impact on pathogen performance at the species level. For this approach, we selected a novel yeast β-glucan as our test fibre and compared it with glucose as a negative control in the presence and absence of a cocktail of pathogens. Fermentation with both glucose and yeast β-glucan reduced alpha and beta diversity indices. A similar phenomenon was observed by Luo *et al.*^[31]^ in mice when oat β-glucan significantly decreased the alpha diversity of the murine gut microbiota, suggesting that a select few taxa can metabolise this substrate. However, when oat β-glucan was mixed with microcellulose, an insoluble dietary fibre, alpha diversity increased due to the presence of increased carbon sources for a greater range of bacterial taxa. In the same study, oat β-glucan increased the relative abundance of *Bacteroides*. Indeed, *Bacteroides* species are among the most prominent fibre-degrading commensals in the gastrointestinal tract, and in this study, we detected eight species of *Bacteroides* in the baseline faecal slurry. Accordingly, we observed that *B. uniformis*, and to a lesser extent, *B. ovatus*, were much higher in the fibre groups compared with the glucose groups. The growth of these two species in the glucose group suggests that the choice of medium may provide an advantage over the other *Bacteroides* species. However, a recent study comparing the degrading capacity of mixed-linkage β-glucans in 121 strains of *Bacteroides* found that only seven strains could grow on β-glucans^[32]^. Interestingly, 34/34 *B. ovatus* and 33/35 *B. uniformis* strains tested in that study were competent β-glucan metabolisers, which would explain the large relative abundances of these species compared with the other *Bacteroides* in the yeast β-glucan groups. This observation further supports the nutrient niche theory, whereby a fibre can target particular species in a complex gut community^[33]^. Two species of Firmicutes performed well in this system: both belonging to the *Clostridium senso stricto* genus. An unclassified *Clostridium sensu stricto* found in low abundance in the initial inoculum was relatively higher in three of the fermentation groups, apart from the GN group, suggesting that the model and the choice of medium itself were well suited to this species. Concentrations of the SCFA butyrate were also much lower in the GN group compared with the others. These observations combined suggest that this unclassified *Clostridium sensu stricto* may be contributing to the production of butyrate. Indeed, it is known that commensal *Clostridia* are among the main producers of butyrate in the gut^[34]^. We observed some species to be significantly higher in the presence of glucose compared with the fibre groups. An unclassified species of *Leuconostoc*, a member of the *Lactobacillales* family, was undetectable in our inoculum samples but was found to be higher in the GN group but not the GP group. The higher relative abundance in this species in the GN group suggests that either the pathogens are exhibiting an antagonistic effect on its growth either by production of antimicrobials or through competition for glucose. Species of *Leuconostoc* have previously been shown to have a high fermentation capacity for glucose under anaerobic conditions^[35]^ compared to other sugars; therefore, it is likely that the pathogens are outcompeting *Leuconostoc* through glucose utilisation. The high concentrations of butyrate in the GP group, but not the GN group, suggest that the pathogens may be contributing to the production of butyrate when anaerobically fermenting glucose. A member of the *Sutterella* genus with homology to *S. stercoricanis*^[36]^ was observed to be elevated in the glucose-treated groups. Members of *Sutterella* have been associated with autism and IBD, yet more recent studies have found that they are abundant in the duodenum of healthy adults with a decreasing gradient toward the colon and display mild-proinflammatory activity^[37]^, indicating they may have an immunomodulatory role. We did not find any significant differences in the relative abundances of pathogens in the fibre or glucose groups.

A drawback of 16S rRNA amplicon sequencing is accurately determining species and strain classification owing to the similarities in the 16S gene, and the depth of taxonomic resolution based on the V3-V4 region, and this is even more apparent when attempting to separate closely related members of the *Enterobacteriaceae,* both in terms of pathogenic and commensal microbiome members*.* Indeed, a recent study demonstrated that choosing the V2-V3 region (205bp) provided higher resolution for lower-rank taxa, i.e., genera and species, compared with the V3-V4 region (443bp)^[38]^. We, therefore, employed qPCR to detect individually spiked pathogens in the *ex vivo* gut model after 24 hours of fermentation to assess the potential putative protective effects of a selection of dietary fibres on pathogen proliferation. Surprisingly, the growth of *E. coli* and *S. typhimurium* was almost completely inhibited in the glucose-treated group following 24 hours of fermentation [Figure 6A and B], suggesting that commensal bacteria in the faecal inoculum suppressed their growth. It has been shown in previous studies that metabolites produced by commensals such as SCFAs can inhibit *Salmonella* growth^[39]^ and slow the growth of T3SS-1 (type-3 secretion system 1) expressing subpopulations^[40]^. It may be possible that glucose-mediated commensal *Enterobacteriaceae* blooms outcompete the spiked pathogenic *Enterobacteriaceae* growth through undetermined mechanisms. A recent study demonstrated that commensal *E. coli* can limit *Salmonella* growth by up to 10,000-fold after challenge in mice compared to a commensal microbiota lacking *E. coli*^[41]^. Conversely, pathogenic *Enterobacteriaceae* growth was not inhibited in the three fibre-treated groups, even though these pathogens were unable to metabolise any of the tested fibres. Fibre-degrading bacteria are primarily members of the *Bacteroides/Prevotella* and *Firmicutes* genus^[42]^, and these may not possess inhibitory properties against pathogenic *Enterobacteriaceae* or have much slower growth kinetics to produce an inhibitory effect.

*E. faecium* strain TX16 was subjected to the same fermentation procedure as *S. typhimurium* and *E. coli*. This strain, isolated from a patient with endocarditis, represents a nosocomial lineage (ST18) responsible for the majority of multidrug-resistant *E. faecium* infections^[43]^. Interestingly, the growth of this pathogen was not inhibited in the glucose group; however, its growth was significantly inhibited in the presence of yeast β-glucan, arabinoxylan, and oat fibre. In fact, a 1-log reduction in growth in the yeast β-glucan group was obtained (*P* < 0.01), suggesting that this fibre may be a candidate for reducing *E. faecium* burden in hospitalised patients*. E. faecium* is a member of the gram-positive Firmicutes phylum, and it may be possible that commensal fibre-degrading Firmicutes outcompete this pathogen’s growth, as demonstrated by a faecal microbiota transplantation (FMT) trial that identified members of the *Lachnospiraceae* and *Clostriaceae* families involved in VRE clearance^[44]^. To our knowledge, there has been limited research conducted on the potential of dietary intervention to control multidrug-resistant *Enterococcus*; however, combining it with FMT to enhance the clearance of multidrug-resistant *Enterococcus* remains an appealing approach.

Unlike the other gastrointestinal pathogens which colonise the colon, *L. monocytogenes* predominantly infects the ileum^[45]^. Dietary fibres that encourage the growth of closely related lactic acid bacteria (LAB) could, in theory, improve colonisation resistance against *L. monocytogenes.* We included an additional test substrate GOS as a comparison to our test fibres, and an additional cellulose control, as GOS has previously been shown to inhibit *L. monocytogenes* colonisation in a guinea pig model^[15]^. Given that L. monocytogenes naturally inhabits the soil, it is possible that it possesses the means to metabolise yeast β-glucan or its metabolites, as the structure of yeast β-glucan resembles that which is found in a range

of fungi^[46]^. Our results demonstrate that yeast β-glucan can promote the growth of *L. monocytogenes*, whereas GOS can suppress growth, reaffirming the findings of an *in vivo* study^[15]^. A recent study found that commensal microbes of the *Clostridiales* order rapidly cleared *L. monocytogenes* from the intestinal lumen of mice^[47]^, and thus identifying prebiotic substrates that enhance the growth of commensals such as *Clostridiales* could help provide dietary-mediated colonisation resistance against *L. monocytogenes*.

Our *ex vivo* model can help identify dietary substrates that may help boost colonisation resistance without the implementation of more expensive *in vivo* animal models. Nonetheless, there are some limitations of our approach that we wish to address. We pooled faecal samples from healthy subjects to circumvent the monetary and human resources that would be required by performing fermentations on individual faecal samples. Thus, only *n* = 1 microbiota was essentially tested. Owing to the large inter-individual microbial variation, we realise that individual microbiotas may respond differently to dietary interventions and thus the perceived effect of colonisation resistance. However, other *in vitro* models have confirmed a functional overlap in microbial activity while studying individual faecal inoculum, despite differences in microbial composition^[48]^, thereby supporting pooling as a representation of a larger population. Moreover, the choice of glucose control as a comparison would not represent a colonic available quantitative substrate and thus may be seen as a limitation. We opted to compare β-glucan to a control of its individual monomers, i.e., glucose, and subsequently added these substrates to a no-carbon medium to examine the effects of these carbohydrates on microbiome composition with and without the addition of pathogens. Additionally, our experiments were completed following 24-hour fermentation, which may only feed the primary degraders of our tested fibres and this timeframe may not be sufficient to feed the carbon trophic chain and, in theory, the high inoculum (5%) only allows for four generations. Thus, we may be missing important taxa that do not have enough time to grow in the 24-hour period. Moreover, following faecal sample collection, we prepared our standardised faecal preparation on the day of collection and this was frozen on the same day. This approach allowed us to prepare our fermentation experiment and pathogen growth, and the standardised faecal sample was thawed on the day of fermentation and quickly added to the fermenter. We realise that the freezing process may compromise some bacterial taxa; however, our procedure of freeze preparation has been demonstrated to have a limited effect on the majority of taxa^[18]^. Finally, quantification of the total microbial communities would provide an important addition, enabling a comprehensive view of the interactions between microbiome, fibres, and pathogens. Our research group is already working on this approach by spiking human microbiome samples with known concentrations of DNA from marine microorganisms not normally present in the human gut microbiome that directly correlate with bacterial cell numbers. This approach, combined with qPCR and sequencing, allows quantification of the absolute abundance of taxa within the microbiome. Using such an approach in this study would provide a better understanding of the impact of the fibres on the microbiome as a whole, and better identify which species are directly influenced by the fibres to provide colonisation resistance against pathogens. Furthermore, the inclusion of shotgun metagenomic sequencing data would enable the identification of genes in taxa involved in fibre degradation and fermentation to further support the quantification data. Flow cytometry, microscopy, and culturing would also aid quantification studies.

In conclusion, the fibres tested in this pilot study differentially impacted the growth of pathogens in an *ex vivo* model of the gut microbiota. However, yeast β-glucan, in particular, could represent a dietary strategy to control VRE expansion in the gut. Thus, yeast β-glucan should be tested against other VRE strains in the future. Moreover, our pilot investigation opens opportunities for a new avenue of research, including the possibility of screening other dietary fibres and oligosaccharides, such as fructo-oligosaccharides (FOS) from plants, as means of boosting colonisation resistance against food-borne pathogens. While the burden of gastrointestinal infections is higher in developing nations as opposed to westernised nations^[49]^, with the former retaining higher fibre intake, the encroaching threat of antibiotic-resistant pathogens in the West prompts the adoption of new, or in essence, old strategies to limit pathogen expansion in the gut. Our findings from this pilot study can provide a platform to explore this paradigm further and inform future animal studies by means of a starting point.

## DECLARATIONS

### Availability of data and materials

Raw 16S rRNA gene sequencing data are available at the NCBI Sequence Read Archive (SRA), under BioProject PRJNA971893.

### Copyright

© The Author(s) 2023.
